# Applications of Bayesian approach in modelling risk of malaria-related hospital mortality

**DOI:** 10.1186/1471-2288-8-6

**Published:** 2008-02-19

**Authors:** Lawrence N Kazembe, Tobias F Chirwa, Jupiter S Simbeye, Jimmy J Namangale

**Affiliations:** 1 Applied Statistics and Epidemiology Research Unit, Mathematical Sciences Department, Chancellor College, University of Malawi, Zomba, Malawi; 2 Malaria Research Programme, Medical Research Council, Durban, South Africa

## Abstract

**Background:**

Malaria is a major public health problem in Malawi, however, quantifying its burden in a population is a challenge. Routine hospital data provide a proxy for measuring the incidence of severe malaria and for crudely estimating morbidity rates. Using such data, this paper proposes a method to describe trends, patterns and factors associated with in-hospital mortality attributed to the disease.

**Methods:**

We develop semiparametric regression models which allow joint analysis of nonlinear effects of calendar time and continuous covariates, spatially structured variation, unstructured heterogeneity, and other fixed covariates. Modelling and inference use the fully Bayesian approach via Markov Chain Monte Carlo (MCMC) simulation techniques. The methodology is applied to analyse data arising from paediatric wards in Zomba district, Malawi, between 2002 and 2003.

**Results and Conclusion:**

We observe that the risk of dying in hospital is lower in the dry season, and for children who travel a distance of less than 5 kms to the hospital, but increases for those who are referred to the hospital. The results also indicate significant differences in both structured and unstructured spatial effects, and the health facility effects reveal considerable differences by type of facility or practice. More importantly, our approach shows non-linearities in the effect of metrical covariates on the probability of dying in hospital. The study emphasizes that the methodological framework used provides a useful tool for analysing the data at hand and of similar structure.

## Background

*Plasmodium falciparum *malaria is a major public health problem in most tropical countries in the world. Between 300 and 500 million cases of clinical episodes occur each year, and 1–3 million people die of the disease [1,2]. The sub-Saharan African region has the greatest burden with over 90% cases and 80% malaria-attributable deaths [3]. Measuring malaria burden in a population is a challenge in most developing countries [1,2], because most disease incidences and deaths occur outside of the formal health care, particularly at home [4,5]. Instead, routine hospital data provide a proxy for measuring the incidence of severe malaria and for crudely estimating morbidity rates or equivalent clinical indicators [6].

Analysis of these data may allow to assess, compare and ultimately improve the care provided at all levels of health care. It may assist in monitoring and planning resource needs in a health system and designing appropriate interventions, tailored towards communities at high risk or lead to further investigations to identify important risk factors [7]. Variability in these indicators is a well known issue, and is a function of various covariates, at both patient or group level, some observed and others unobserved, and maybe spatially correlated or time-varying [7-10]. Geographical differences are driven by socio-economic determinants, availability and access to health care or health seeking behaviour [7,10]. Temporal variation may again be a factor of access to care and malaria transmission [9], for example, there can be increased access in dry season and yet fewer cases in the same season. Adequate statistical modelling and analysis is, therefore, of epidemiological interest.

This paper is motivated by the analysis of malaria-related hospital mortality data collected at patient's level, covering a period of two years among children admitted to a referral district hospital in Malawi. The response variable is binary (whether died of malaria in hospital or not) and is linked to several covariates which are categorical or continuous, spatial and temporal. Unobserved heterogeneity due to, for example, differences in practice style or type of hospital, inequalities in utilisation or access, may exist and should be explored. Hierarchical regression modelling provides a general framework to investigate the effect of these cofactors.

We apply a geoadditive logistic model as proposed by Fahrmeir and Lang [11]. Applications of such models are many and literature is growing. These models can be estimated through a fully or empirical Bayesian approach, and are implemented in BayesX [12]. For example, Augustin et al. [13] employed the model to study the relationship between needle losses of pine-trees and various covariates. Inference was performed with a full Bayes (FB) approach making use of Markov Chain Monte Carlo (MCMC) simulation techniques. Tutz [14] developed a class of generalised semiparametric mixed models and proposed penalized marginal likelihood approach for the estimation of parameters. Fahrmeir et al. [15] considered a penalised geoadditive model for space-time data with inference performed using an empirical Bayesian (EB) approach.

In this paper, we use the fully Bayesian approach via MCMC simulation techniques. The advantages of FB inference is that the functionals of the posterior can be computed without relying on large sample Gaussian justifications, and the approach is computationally feasible for large datasets. Moreover, the uncertainty in the parameters is easily quantified [15]. Furthermore, Bayesian methods are more flexible in that empirical information, when available, can be incorporated with the data through an informative prior distribution. When this information is not available, a non-informative prior can be chosen. The methodology is of substantive interest since the effects of other covariates are jointly estimated with the random effects, e.g., spatially structured and unstructured heterogeneity effects [16]. This is extended to incorporate nonparametric terms for nonlinear continuous covariates and time-varying coefficients, for example, time trend and seasonal variation of calendar time. In addition, space-time interactions are assessed within the varying-coefficient models framework [17].

The rest of this paper is organised as follows. We first describe the data. Next, we specify the model and outline the Bayesian approach used for model estimation. This is followed by the application of the model to the data, and then results are presented. Discussion on the results and limitations of the study conclude the article.

## Methods

### Data

Data were obtained from discharge records of all paediatric hospital admissions at Zomba district hospital, Malawi, between 1 January 2002 to 31 December 2003. Each case was confirmed as malaria on admission through microscopic identification of parasites in blood samples. Zomba district hospital, with over 500 beds is the largest health facility in the district and serves both as the first consultation point for patients within its catchment, and as a referral centre for other 23 primary health centres. These facilities are managed by the Ministry of Health and the Christian health association of Malawi, and variations in health care management is expected.

The discharge registers included patients' age, sex, date of admission and discharge, whether referred to the hospital or not, the discharge outcome (i.e. death, discharged home, home-based care or absconded), village or location of residence, and treatment given. Based on the name of the village, each case was matched to one of 21 residential wards in the district. Approximately 86% of cases were successfully linked to wards, the other 14% having either missing or insufficient residential information. Only geo-referenced cases are included in this analysis. Table 1 gives a description of the variables used in this analysis.

**Table 1:** Descriptive summary of variables used in the study.

	Description	*n *(per cent)^§^
Binary variables		

Sex	1 = female	1683 (7.7)
	0 = otherwise	2286 (7.6)
Day	1 = if admitted over weekend	2418 (7.5)
	0 = otherwise	1492 (7.5)
Season	1 = if admitted during dry season	1128 (5.4)
	0 = otherwise	1128 (5.4)
Distance	1 = if distance travelled is ≤ 5 km	1938 (7.4)
	0 = otherwise	1999 (8.8)
Referral	1 = if referred to hospital from networking PHC	1895 (8.8)
	0 = otherwise	1494 (6.1)
		
Metrical variable		Mean (SD^‡^)

age	Age of child	30.5 (30.7)
los	Length of hospital stay	78.9 (264.1)
ct	Calendar time	44.8 (30.1)
		
Spatial/heterogeneity variables		

*v*	21 structured residential wards effects	
*u*	21 unstructured residential wards effects	
*h*	23 unstructured primary health care (PHC) facility effects	
*N*	Total number of observations	3969

^§^*n *= number hospitalised and percent died in that category

^‡^SD = standard deviation

A total of 302 deaths were registered among 3,969 children hospitalised for malaria, between January 2002 to December 2003, resulting in an overall case fatality ratio (CFR) of 7.6%. Table 1 shows the proportion who died in different covariate levels. The proportion varies with age, referral status, season, distance from the hospital and length of hospital stay (LOS). The CFR drops from 8.5% in the age of *<*1 year to 6.2% at age of between 1–4 years and increases in the 5–14 years groups to 10.5. This suggests a curvature in the association of age and the probability of in-hospital mortality. The number of cases are relatively more in the wet season (October-March) compared to the dry season (April-September), with a similar pattern of CFR. Boys are more frequently hospitalised than girls (58%), but the CFR is not different. The hospital receives relatively more patients from a distance of more than 5 kms (52%), with distant patient likely to die in hospital. As for LOS, CFR is very high on day 1, drops and then increases as the stay is prolonged. Again there is an indication of curvature in the relationship between LOS and the risk of inpatient mortality. Children referred to the hospital are most likely to die in hospital (CFR = 8.8%). Further detailed descriptive and exploratory analyses presented elsewhere clearly show spatial and temporal variations [10].

### The Model

Given a set of observations (*y*_*i*_, ***w***_*i*_), *i *= 1, ⋯, *n*, where *y*_*i *_is a binary response such that *y*_*i *_= 1 if a child died in hospital and *y*_*i *_= 0 a child is discharged, and ***w***_*i *_= (*w*_*i*1_, ⋯, *w*_*ip*_)' are covariates, we consider a logistic model to estimate the probability of dying in hospital, *y*_*i *_= 1 versus the probability of being discharged from hospital, *y*_*i *_= 0. The response is distributed as a Bernoulli random variable such that:

$$f(y_{i}|\eta_{i})=p_{i}^{y_{i}}{\left(1-p_{i}\right)}^{1-y_{i}}=\exp[y_{i}\eta_{i}-\log(1+\exp(\eta_{i}))]$$

where *p*_*i *_= *P*(*y*_*i *_= 1), and *η*_*i *_= logit(*p*_*i*_) is a canonical parameter linked to the linear predictor

$$\eta_{i}={w^{\prime }}_{i}\gamma .$$

Here *γ *is a *p*-dimensional vector of unknown regression coefficients.

Since the observations are associated with location of residence, it is desirable to account for geographical differences. We introduce areal level effects to allow expected spatial correlation and any unstructured areal heterogeneity of morbidity, using a convolution prior [16]. We also specify health facility effects, which permit variations that occur by type of facility. These supply effects may impact on the referral patterns, admission patterns and case management. Furthermore, we assume additional flexibility in the predictor to allow for nonlinear or time-varying covariate effects. We, therefore, extend the predictor (2) to a more general semiparametric predictor [11],

$$\eta_{i}=v_{i}+u_{i}+h_{i}+f_{1}(x_{i})+f_{2}(t_{i})+{w^{\prime }}_{i}\gamma$$

where *v*_*i*_, *v *∈ {1, ⋯, *V*} are spatially structured effects for child *i*; *u*_*i*_, *u *∈ {1, ⋯, *U*} and *h*_*i*_, *h *∈ {1, ⋯, *H*} model unstructured heterogeneity at area and health facility levels respectively, *f*_*i *_are unknown functions for nonlinear effects of continuous covariate *x*_*i *_(e.g., age of the child), or calendar time effect *t*_*i*_. Note that the spatially structured effects and unobserved heterogeneity tries to capture all sources of unmeasured influential factors, some that occur locally or at large scale, or those that may vary with time.

Several extensions to the additive predictor (3) are possible. For example, the calendar effect *f*(*t*_*i*_) can be decomposed into time trend *f*(*r*_*i*_) and time-varying seasonal component *f*(*s*_*i*_), i.e.,

*η*_*i *_= ⋯ + *f*(*r*_*i*_) + *f*(*s*_*i*_) + ⋯,

In addition, the model can be extended to include interaction surfaces within the varying coefficient framework proposed by Hastie and Tibshirani [17]. Here the effect of some covariate *z *is assumed to vary smoothly over the range of a second covariate *x*, giving the predictor

*η*_*i *_= ⋯ + *f*(*x*_*i*_)*z*_*i *_+ ⋯,

of which the term *f*(*x*)*z *= *g*(*x*, *z*) is interpreted as an interaction term between *z *and *x*. In our case study, this can be time-space interactions, leading to a predictor of the form

$$\eta_{i}=v_{i}+u_{i}+h_{i}+f_{1}(x_{i})+f_{2}(r_{i})+f_{3}(s_{i})+f_{4}(v_{i})r_{i}+{w^{\prime }}_{i}\gamma .$$

The function *f*_4 _quantifies the deviations from the effect at some specified reference or baseline time period. This will be discussed in detail in a separate analysis.

### Estimation: fully Bayesian approach

#### Prior distributions for covariate effects

Modelling and inference uses the fully Bayesian approach. In the Bayesian formulation, the speci-fication of the proposed model (Equation 4) is complete by assigning priors to all unknown parameters. For the fixed regression parameters, a suitable choice is the diffuse prior, i.e., *p*(*γ*) ∝ *const*, but a weakly informative Gaussian prior is also possible. For the time and continuous covariates we estimate them nonparametrically through smoothness priors. We use the second-order Gaussian random walk prior to allow enough flexibility, while penalising abrupt changes in the function, as suggested by Lang and Brezger [18]. The prior can be expressed in the pairwise difference form as

$$p(f|\tau_{f}^{2})\propto \exp\left(-\frac{\tau_{f}^{2}}{2}\sum_{t=3}^{T}{\left(f_{t}-2f_{t-1}+f_{t-2}\right)}^{2}\right)$$

where ***f ***= (*f*_1_, ⋯, *f*_*p*_) and $\tau_{f}^{2}$ is the variance, with diffuse priors *f*_1 _∝ *const*, *f*_2 _∝ *const *for initial values.

For the time-varying seasonal effect, we also assign a smoothness prior whose joint distribution, ***s***, is given by

$$p(s|\tau_{s}^{2})\propto \exp\left(-\frac{\tau_{s}^{2}}{2}\sum_{t=12}^{T}{\left(s_{t-11}+\cdots +s_{t}\right)}^{2}\right),$$

again assuming diffuse priors for initial values, *s*_1_, ⋯, *s*_11_, and $\tau_{s}^{2}$ is a variance that controls the degree of smoothness. The unstructured spatial heterogeneity term, *u*_*i *_is assumed to follow an exchangeable Gaussian prior with zero mean and variance, $\tau_{u}^{2},\text{i}.\text{e}.,u_{i}\sim N(0,\tau_{u}^{2})$. A similar prior is assigned to the heterogeneity term for the health facility, i.e., $h_{i}\sim N(0,\tau_{h}^{2})$.

Finally, for the spatial components *v*_*i*_, we assign a Markov random field (MRF) prior [16]. This is analogous to random walk models. The conditional distribution of *v*_*i*_, given adjacent areas *v*_*j*_, is a univariate normal distribution with mean equal the average *v*_*j *_values of *v*_*i*_'s neighbouring areas and variance equal to $\tau_{v}^{2}$ divided by the number of adjacent areas. This leads to a joint density of the form

$$p(v|\tau_{v}^{2})\propto \exp\left(-\frac{\tau_{v}^{2}}{2}\sum_{i\sim j}{\left(v_{i}-v_{j}\right)}^{2}\right)$$

where *i *~ *j *denotes that area *i *is adjacent to *j*, and assumes that parameter values *v*_*i *_and *v*_*j *_in adjacent areas are similar. The degree of similarity is determined by the unknown precision parameter $\tau_{v}^{2}$.

By writing ***f***_*j *_= ***Z***_*j*_***β***_*j*_, ***h ***= ***Z***_*k*_***β***_*k*_, ***u ***= ***Z***_*l*_***β***_*l *_and ***v ***= ***Z***_*m*_***β***_*m*_, for a well defined design matrix ***Z ***and a (possibly high-dimensional) vector of regression parameters ***β***, all different priors (Equations 5–7) can be expressed in a general Gaussian form

$$p(\beta_{j}|\tau_{j}^{2})\propto \exp\left(-\frac{1}{2\tau_{j}^{2}}{\beta^{\prime }}_{j}K_{j}\beta_{j}\right)$$

with an appropriate penalty matrix ***K***_*j*_. Its structure depends on the covariate and smoothness of the function. In most cases, ***K***_*j *_is rank deficient and hence the prior for ***β***_*j *_is improper. For the variances $\tau_{j}^{2}$ we assume inverse Gamma priors *IG*(*a*_*j*_, *b*_*j*_), with hyperparameters *a*_*j*_, *b*_*j *_chosen such that this prior is weakly informative.

### Posterior distribution

Fully Bayesian inference is based on the analysis of posterior distribution of the model parameters. In general the posterior is highly dimensional and analytically intractable, which makes direct inference almost impossible. This problem is circumvented by using MCMC simulation techniques, whereby samples are drawn from the full conditional of parameters given the rest of the data. Under conditional independence assumptions the posterior distribution for the Bernoulli model is given by Bayes Theorem

$$\begin{array}{lll} p(\beta ,\tau^{2},\gamma |data) & \propto & L(data|\beta ,\tau^{2},\gamma )p(\beta ,\tau^{2},\gamma )\\ & = & L(data|\beta ,\tau^{2},\gamma )\\ & \times & \left\{\prod_{i=1}^{p}p(\beta_{j}|\tau_{j}^{2})p(\tau_{j}^{2})\right\}p(\gamma ) \end{array}$$

where the quantity *p*(***β***, ***γ***, ***τ***^2^) is the prior density function, and *L*(*data*|***β***, ***γ***, ***τ***^2^) denotes the likelihood of the Bernoulli model. More specifically, the posterior is given by

$$\begin{array}{lll} p(\beta ,\tau^{2},\gamma |data) & \propto & \prod_{i=1}^{n}p_{i}^{y_{i}}{\left(1-p_{i}\right)}^{1-y_{i}}\\ & \times & \exp\left\{-\frac{1}{2\tau_{j}^{2}}{\beta^{\prime }}_{j}K_{j}\beta_{j}\right\}\\ & \times & \prod_{j=1}^{k}\frac{1}{\Gamma (a_{j})b_{j}^{a_{j}}}{\left(\tau_{j}^{2}\right)}^{-(a_{j}+1)}\exp(-\frac{b_{j}}{\tau_{j}^{2}})\times p(\gamma ). \end{array}$$

For updating the full conditionals of parameters, we use a hybrid MCMC sampling scheme of the iteratively weighted least squares (IWLS) proposals, developed for generalised linear mixed models by Gamerman [19], and Metropolis-Hastings algorithm. Full details are presented elsewhere [11,14,15,18].

### Applications

We analyse the following logistic models,

M0: *η*_*i *_= ${w^{\prime }}_{i}\gamma$

M1: *η*_*i *_= ${w^{\prime }}_{i}\gamma$ + *f*_1_(*age*) + *f*_2_(*los*) + *f*_3_(*ct*)

M2: *η*_*i *_= ${w^{\prime }}_{i}\gamma$ + *f*_1_(*age*) + *f*_2_(*los*) + *f*_3_(*ct*) + *v*_*i *_+ *u*_*i *_+ *h*_*i*_

M3: *η*_*i *_= ${w^{\prime }}_{i}\gamma$ + *f*_1_(*age*) + *f*_2_(*los*) + *f*_3_(*trend*) + *f*_4_(*season*) + *v*_*i *_+ *u*_*i *_+ *h*_*i*_

Model M0 is a basic regression model of fixed covariates only (Table 1). Model M1 assumes nonlinear functions for the continuous factors, i.e., age *f*_1_(*age*), and LOS *f*_2_(*los*) and calendar time *f*_3_(*ct*) measured in weeks, and tries to assess the gains of fitting a semiparametric model. The choice of estimating age and LOS using nonlinear smoothing priors is motivated by preliminary results, see Ref. [10], which clearly suggest a nonlinear relationship in LOS and possibly one in age. Model M2 considers all possible risk factors, i.e., we simultaneously analyse nonlinear effects of age, time trend of calendar time, structured spatial effects, *v*, for the 21 residential wards, unstructured spatial effects, *u*, heterogeneity effects, *h*, for the 23 health facility, and fixed effects, ***w'γ***, for the categorical variables. In model M3, we extend model M2 to consider further temporal effects, whereby the effect of calendar time is decomposed into a time trend, *f*_3_(*trend*) and seasonal component, *f*_4_(*season*).

We implement the models in BayesX ver 1.4 – a public domain software for computing complex Bayesian techniques [12]. For the four models, 40,000 iterations are carried out after a burn-in sample of 10,000. We thin every 20th iteration, yielding 2,000 samples for parameter estimation. Convergence is monitored by plotting trace and autocorrelation plots of the samples. Quantiles, median, mean and standard deviation for all parameters, estimated from the posterior distributions, are used to assess model fit. In particular, credible intervals are used to assess the significance of parameters.

We also monitored the posterior deviance, and compared the set of plausible models using the Deviance Information Criterion (DIC) [20]. Specifically, we compare the structured additive models (i.e., M1, M2 and M3) with the simpler parametric alternative (M0). The DIC is given by *DIC *= $\bar{D}$ + *p*_*D*_, where $\bar{D}$ is the posterior mean of the deviance, which is a measure of goodness of fit, and *p*_*D *_is the effective number of parameters, which is a measure of model complexity and penalises overfitting. Since small values of $\bar{D}$ indicate good fit while small values of *p*_*D *_indicate a parsimonious model, small values of DIC indicate a better model. Models with differences in DIC of *<*3 compared with the best model can not be distinguished, while those between 3–7 can be weakly differentiated [[24], p.613].

## Results

### Model assessment

Comparing the goodness of fit of models M0, M1, M2, and M3 we note that M3 is a preferred model (Table 2). The difference between model M3 and model M0 is Δ*DIC *= 661:01. Note that models M1 and M2 are also better fitting than the basic model M0 with *DIC *= 1372:39. Indeed, assuming a semiparametric model slightly improved the model fit compared to estimating a fully parametric model (*DIC *= 1372:39 in M0 versus *DIC *= 1369:06 in M1, Δ*DIC *= 3:33). The inclusion of random effects further improves the model fitness despite increased model complexity (*DIC *= 1369:06 in M1 versus *DIC *= 729:07 in M2, Δ*DIC *= 639:99). Evidently, modelling the impact of known factors alone is not sufficient to produce a satisfactory fit to the observations, and random effects at area and health care level are needed to improve fit and account for heterogeneity. In our analysis, we also observe that the inclusion of random effects reduce the effect size of some variable (results not shown). In what follow, we only report results based on model M3.

**Table 2:** Comparison of the four fitted models using the Deviance information criteria. See text for details.

	Models			
	*M*0	*M*1	*M*2	*M*3
*Model fit*				
$\bar{D}$	1360.41	1347.58	684.75	651.54
*p*_*D*_	11.98	21.48	61.01	59.84
*DIC*	1372.39	1369.06	729.07	711.38
Δ*DIC*^§^	661.01	657.68	17.69	0

^§ ^Difference of the best model M3 against others

### Fixed effects

Table 3 gives posterior means and odds ratios (OR), and the corresponding 95% credible interval (CI) for categorical covariates. The risk of dying in hospital is related to season, distance to the hospital and referral status of a child. No association is observed between probability of dying in hospital and sex, nor between probability of dying and day of the week. The likelihood of dying in hospital is lower in the dry season relative to the wet season (OR: 0.63, 95% CI: 0.49 to 0.86). For children who travel less than 5 kms to the hospital compare to those who travel more than 5 kms, the risk of dying in hospital is lower (OR: 0.005, 95% interval: 0.0006 to 0.28). Children referred to the hospital are at increased risk of dying in the hospital relative to those who do not (OR: 98.49, 95% interval: 21.33 to 383.75).

**Table 3:** Estimates of fixed parameters based on Model *M*3.

		Model coefficients		Odds Ratio	
Covariate		Mean^‡^	95% CI^§^	Mean	95% CI
Sex	Female child	-0.09	-0.35, 0.14	0.92	0.79, 1.19
	Male	0		1.00	
Day	Weekend	0.19	-0.06, 0.38	1.19	0.94, 1.46
	Weekday	0		1.00	
Season	Wet	0		1.00	
	Dry	-0.48	-0.77, -0.24	**0.63**	**0.49, 0.86**
Distance	≤ 5 kms	-5.15	-7.73, -2.60	**0.005**	**0.0006, 0.28**
	> 5 kms	0		1.00	
Referral	Yes	4.59	3.45, 5.85	**98.49**	**21.33, 383.75**
	No	0		1.00	

^§ ^CI = Credible interval; ^‡^Posterior mean

### Nonlinear effects

**Figure 1:**
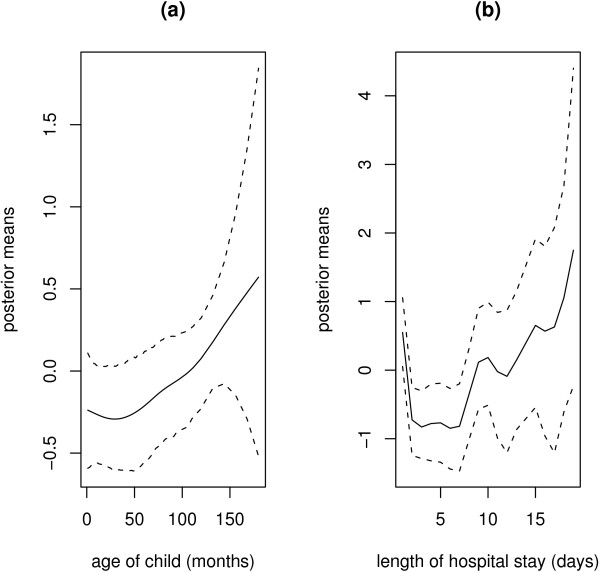
Nonlinear effect of (a) age of the child (in months); (b) length of hospital stay (in days). Shown are the posterior means (solid line) together with 95% pointwise credible intervals (dotted line).

Figure 1 displays the nonlinear effects of age of child and LOS on the probability of dying in hospital. The effect of age is estimated to be almost linear, with the posterior means increasing with increasing age (Figure 1a). In other words the risk is lower for infants, but increases for much older children. For LOS, the posterior means show slight deviation from linearity (Figure 1b). The risk decreases from day 1, remains almost constant from day 2 to 6, and then increases from day 7 to 20.

### Temporal effects

**Figure 2:**
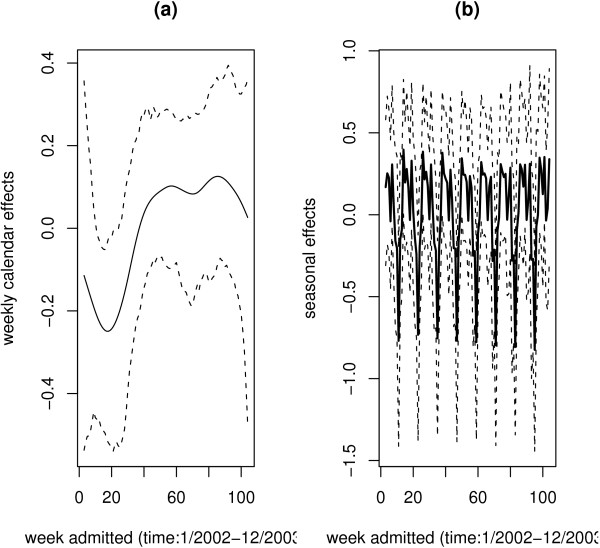
Temporal variation of risk: (a) time trend, and (b) seasonal effect at time of admission (in weeks). The posterior means (solid line) are plotted together with 95% pointwise credible intervals (dotted line).

Figure 2 displays the temporal effect as measured by the calendar effect. Again the time trend is estimated to be nonlinear (Figure 2a). From week 1 to week 15, the risk decreases, then starts to increase up to week 55. From week 56 the risk is almost constant. The trend closely mirrored wet and dry seasons in the area, with high risk in the rain season and low risk in the dry season. This should be explained by the large number of children hospitalised during the wet season. The seasonal effect is given in Figure 2b. There is a clear seasonal variation for the entire study period. It is evident that the risk pattern displays both within month and between month variability.

### Spatial effects

**Figure 3:**
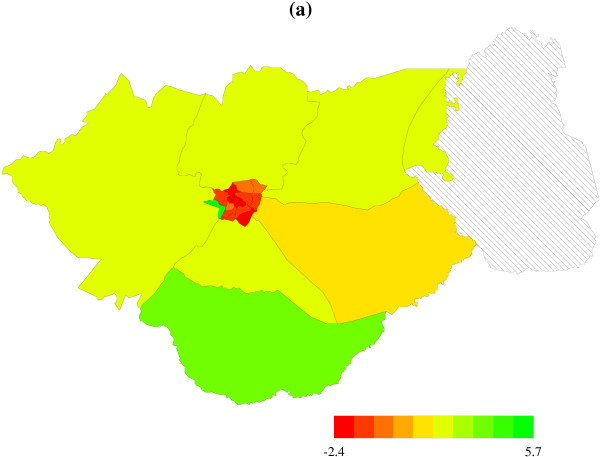
Residual spatial effect of 'residential ward' in Zomba district. Shown are the posterior means. Red colour denotes regions with negative risk, green denotes regions with positive risk. Lake Chilwa is in diagonal solid lines.

**Figure 4:**
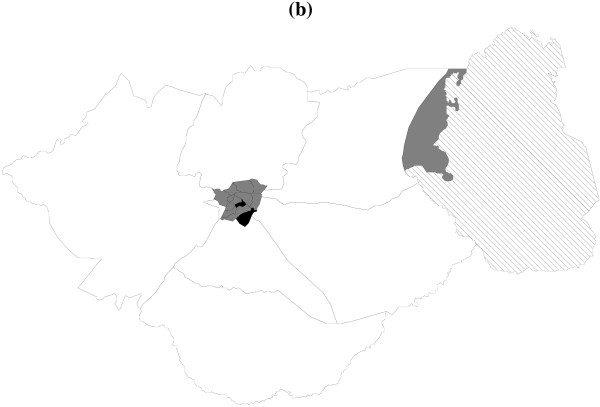
Posterior probabilities, at nominal level of 80%, for the spatial effects in Figure 3. Black denotes regions with strictly negative credible intervals, white denotes regions with strictly positive credible intervals, while grey shows areas of no significant difference. Lake Chilwa is in diagonal solid lines.

**Figure 5:**
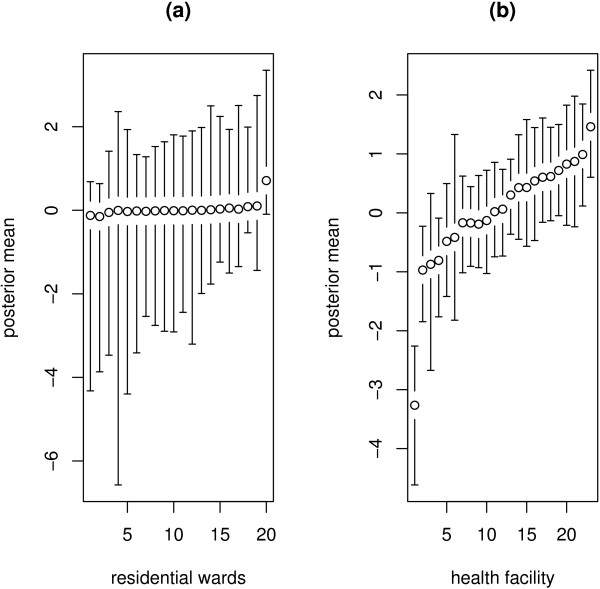
Residual unstructured heterogeneity effects of (a) residential wards, and (b) primary health care facilities. Shown are the caterpillar plots of posterior means (circles), with 95% error bars.

Figure 3 shows the spatial effects with the corresponding posterior probabilities map at 80% nominal level (Figure 4). Areas shade black show strictly negative credible intervals, while white areas depict strictly positive credible intervals, and grey areas indicate nonsignificant credible intervals. There is evidence of spatial variation in risk of dying in hospital. It is clear that areas at the center of Zomba district, which is urban, report reduced risk, while those in the peripheral have increased risk. The uncorrelated spatial heterogeneity is given by caterpillar plot in Figure 5a. There are no clear differences in area specific effects, and most of them have a near zero effect on the probability of dying in hospital. It is clear that the spatially correlated effects are dominant, based on the ratio of variance components, $\varphi =\tau_{v}^{2}/(\tau_{v}^{2}+\tau_{u}^{2})=26.20/(26.20+2.26)=0.92$ (Table 4). The other plot (Figure 5b) displays the heterogeneity effects at health facility level. We observe strong evidence of variation in risk possibly due to differences in health care management at various facilities.

**Table 4:** Sensitivity analysis of model *M*3. Relative changes of fixed effects, deviance information criterion, and variance components for different choices of hyperparameters for $\tau_{v}^{2}$, $\tau_{u}^{2}$ and $\tau_{h}^{2}$.

	Hyperparameters for $\tau_{v}^{2}$, $\tau_{u}^{2}$ and $\tau_{h}^{2}$			
	*a *= 0.5, *b *= 0.0005	*a *= 1, *b *= 0.005	*a *= 0.001, *b *= 0.001	*a *= 0.01, *b *= 0.01
*Model fit*				
$\bar{D}$	651.54	650.12	652.66	650.06
*p*_*D*_	59.84	61.01	61.26	61.53
*DIC*	711.38	711.13	713.92	711.59

*Fixed effects*				
Intercept^§^	-2.78 (-4.04, -1.39)	-3.11 (-4.58, -1.69)	-3.15 (-4.88, -1.43)	-3.16 (-4.72, -1.87)
Sex (female)	-0.09 (-0.35, 0.14)	-0.07 (-0.32, 0.18)	-0.08 (-0.34, 0.17)	-0.08 (-0.32, 0.10)
Season (dry)	-0.48 (-0.77, -0.24)	-0.46 (-0.77, -0.14)	-0.45 (-0.74, -0.18)	-0.48 (-0.77, -0.22)
Distance (≤ 5 km)	-5.15 (-7.73, -2.60)	-5.21 (-8.34, -2.49)	-5.19 (-8.36, -2.22)	-5.17 (-10.02, -1.62)
Referral (yes)	4.59 (3.45, 5.88)	4.61 (3.30, 6.37)	4.51 (3.54, 6.48)	4.65 (3.17, 6.65)
Day (weekend)	0.19 (-0.06, 0.38)	0.20 (-0.01, 0.41)	0.20 (-0.03, 0.43)	0.19 (-0.04, 0.40)

*Random effects*^†^				
Areal: structured ($\tau_{v}^{2}$)	26.27 (10.86, 58.77)	26.58 (12.18, 69.59)	26.44 (8.86, 57.28)	27.34 (11.33, 72.11)
Areal: unstructured ($\tau_{u}^{2}$)	2.26 (0.003, 9.23)	2.19 (0.008, 2.11)	2.34 (0.008, 6.93)	2.22 (0.008, 2.27)
HF: heterogeneity ($\tau_{h}^{2}$)	1.21 (0.48, 2.69)	1.24 (0.43, 2.82)	1.23 (0.29, 1.84)	1.24 (0.46, 3.80)

^§^Posterior mean and 95% credible intervals are given; HF = Health facility

^†^Variance components for the spatially structured effects $\tau_{v}^{2}$, unstructured spatial effects $\tau_{u}^{2}$, and health care random effects $\tau_{h}^{2}$

### Sensitivity analysis

Table 4 reports on the results investigating the influence of hyperpriors since the performance of the model can be sensitive to the choice of the variance components priors [21]. We therefore consider alternative specifications, and carry out sensitivity of our model assuming an IG with scale and shape parameters *a *and *b *respectively. We assume four alternatives *a *= 0.5, *b *= 0.0005, *a *= 1, *b *= 0.005, *a *= 0.001, *b *= 0.001 and *a *= 0.01, *b *= 0.01. The first specification was suggested by Kelsall and Wakefield [22], for modelling the precision of the spatial effects in an MRF model. The second alternative was proposed in Besag and Kooperberg [23]. The remaining two priors with equal scale and shape parameters, especially *a *= *b *= 0.001, have often been used as standard choice on the variances of random effects [24]. Re-running MCMC simulations based on these specifications, using model M3 for simplicity, yield relatively similar inference on risks of dying in hospital, variance components and model fit. Therefore our choice of *IG*(*a *= 0.5, *b *= 0.0005) is appropriate for all the analyses.

## Discussion

This study apply Bayesian techniques to analyse patterns and risk factors of malaria attributable case fatality data. We develop and use logistic regression models to have an in-depth understanding of factors associated with the probability of dying of malaria in hospital, building on the existing methodological contributions by Fahrmeir and Lang [11], Fahrmeir et al. [15].

A number of variables are used to explain the variation in the response and include spatial, continuous, categorical, and heterogeneity terms. The spatially structured variation and unstructured heterogeneity are modelled using MRF prior and zero mean Gaussian heterogeneity priors as proposed by Besag et al. [16]. The continuous variables are estimated non-parametrically by applying second order Gaussian random walk prior, which permits enough flexibility while avoiding over-fitting the data [18]. The proposed methodology allows all these factors to be estimated in a single framework. Because the models are highly parameterised and analytically intractable, the maximum likelihood approach is not be feasible. Thus, the Bayesian inference, making use of MCMC simulation techniques, offers a viable alternative.

In this paper we find evidence that the risk of dying in hospital due to malaria is lower in the dry season, and for children travelling less than 5 kms to the hospital. However, for those referred to the hospital the risk increases. These results seem to suggest that when health care is accessible or available lives can be saved. Malaria is a preventable disease, but delayed treatment or lack of effective treatment can lead to fatal malaria within days [1]. Children are particularly vulnerable because of lack of immunity against the disease [2]. The risk decreases with age, again infants being the most vulnerable, but overall children under five years are the most at risk. The increase in risk for those aged 6–14 years, although these are supposed to be protected through acquired immunity, may reflect some aspects of health seeking behaviour, and emphasize the need for prompt and effective management of malaria for all children including those aged over five years even if such cases may not frequently occur in the general population [7,9,10].

The lower risk in the dry season should be interpreted with care. While the risk of infection is reduced during this period, this effect is directly linked to few cases being hospitalised, hence fewer deaths, and if anything such death should reflect disease management other than severity of the disease. Another possible explanation is that during the dry season access to the hospital is easier than during rainy season, leading to early treatment, and therefore fewer avoidable deaths. Referral children are at increased risk because probably these are already worse-off when they arrive at the hospital. Disease management differences or inaccessibility of care may contribute towards this finding.

The spatial effects are often a surrogate of underlying unobserved information, and may give leads for further epidemiological research or assist in designing malaria interventions. For example, the increased risk in rural areas may be an influence of different factors, such as unavailability or inaccessibility of health facilities resulting in increased risk for such children. These effects may also reflect health seeking behaviour, which plays a critical role in accessing prompt and effective care. Since most antimalarial remedies at first taken at home, effective care may be delayed, leading to increased risk for rural children. Scaling-up of interventions such as insecticide-treated nets or health promotions on appropriate and effective treatment in home or community based care should be emphasized in rural areas [25].

The significance of health facility effects further suggests that management of health care differs in the 23 referring facilities in Zomba district. Indeed, as these are public or private operated, resources such as drugs or ambulatory support may be lacking mainly in government-run health centers. Moreover, some facilities, for example, dispensaries and clinics have limited capacity to treat severe malaria, and may not refer severely sick in time because of lack of communication. There is need to ascertain actual factors contributing to such discrepancy, e.g. using health facility surveys on malaria case management. If indeed, these are the underlying factors, resources need to be committed to improve primary health care. The seasonal variations indicate that malaria transmission processes may explain the variation in the probability of dying in hospital. This is because malaria transmission is highly seasonal and may change within the same area as the year progresses. Essentially, interventions or health promotion campaigns should be tailored in recognition of these varying risk patterns.

The data-driven approach we have taken in this analysis has a greater advantage in that the nonlinear effects of continuous variables are estimated, and avoids ad hoc categorizations although the effect of age can as well be estimated as linear (Figure 1). Indeed, the methodological framework we have applied provide useful tools for handling this type of data, and in similar conditions. Our application demonstrates that spatial and temporal analysis may reveal some salient features of the data, which may be overstepped by the classical regression models (Model M0) or the purely spatial models (Model M2). Flexible modelling, via nonparametric or semiparametric model enable us establish a better epidemiological relationship existing between the response and continuous explanatory variables.

Model selection in this paper is based on the DIC, which is a Bayesian analogue to the Akaike Information Criterion. Although the DIC is now widely used for model choice in complex hierarchical Bayesian models, its usage is at least debatable [[24], pp.612-633]. The DIC measures only the relative goodness of fit among a collection of models. It does not provide information on the adequacy of the model. A model diagnostic tool based on the posterior predictive distribution can be used to assess model adequacy by comparing the observed data with the samples drawn from the posterior predictive distribution. Different approaches for validation, when DIC is not appropriate for decision making on which model to choose have already been employed, for example see Gosoniu et al [26]. Nevertheless, some simulation results [27] suggest that the DIC gives reasonable results even in complex nonparametric regression models.

A major limitation of our analysis is that data used comes from hospital registers. In most African countries, most malaria cases occur at home, and the pattern may be biased towards urban areas that are well covered by health facilities. Moreover, one may argue that much of this data represent severe forms of malaria, because studies on health seeking behaviour for malaria report that biomedical care is sought when the disease is nearly fatal [5]. Health facility data can best be described as providing proxies for prevalence or morbidity and hence health need. A more representative data is through cross-sectional household surveys, e.g. the demographic and health surveys (DHS), however, these are often carried out every four years, thus the periodicity is not frequent enough for surveillance and to inform immediate decision making [4].

## Conclusion

In many resource-poor African countries, collection of population-based health data is a challenge and hospital data provide a critical source of information for decision making. In this paper, we set out to analyse risk factors of malaria mortality, using hospital register data. Our model, using the Bayesian approach, shows that malaria mortality is associated with both individual and group level factors, as well as observed and unobserved risk factors, some of which exhibit spatial and seasonal variation. From a public health perspective, with a goal of prevention and control, our results highlight that reducing malaria burden may require integrated strategies encompassing improved availability and access to effective care at primary facilities; reinforcing home and community case management where prompt care is inaccessible, and encouraging early referral, as well as inducting health promotion interventions aimed at interrupting malaria transmission. Methodologically, this model can easily be adapted to analyse other health indicator of similar structure and in like settings.

## Competing interests

The author(s) declare that they have no competing interests.

## Authors' contributions

LNK conceptualized, analyzed and drafted the manuscript. TFC, JSS and JJN participated in the conception, and critical review of the manuscript. All authors read and approved the final manuscript.

## Pre-publication history

The pre-publication history for this paper can be accessed here:


